# Avoiding touching until 60 min—contamination of transdermal estradiol gel after physical contact

**DOI:** 10.3389/fendo.2025.1524870

**Published:** 2025-06-10

**Authors:** Yi Cao, Le Zhang, Jing Wei, Jingnan Liao

**Affiliations:** Hunan Provincial Key Laboratory of Regional Hereditary Birth Defects Prevention and Control, Changsha Hospital for Maternal & Child Health Care Affiliated to Hunan Normal University, Changsha, China

**Keywords:** case-control study contamination, estradiol gel, menopause hormone therapy, contamination, precocious puberty

## Abstract

**Introduction:**

This study aimed to explore the relationship between the timing of physical contact and the level of estradiol contamination in the skin after application of estradiol gel. Estradiol gel is a common medication used in menopausal hormone therapy (MHT) and understanding its potential for contamination is crucial for ensuring patient safety and effective treatment.

**Purpose:**

The purpose of this hospital-based case–control study was to determine the correlation between the timing of physical contact and the degree of estradiol contamination following the administration of estradiol gel. This information is vital for advising patients on appropriate precautions to minimize the risk of estradiol transfer to others.

**Methods:**

This study was conducted in the gynecology department of Changsha Hospital for Maternal & Child Health Care Affiliated to Hunan Normal University between 2021 and 2022. The participants included 40 menopausal women aged 40–60 years who required MHT and 40 women who did not use estradiol. The intervention involved physical contact after the administration of estradiol gel, and the main outcome measure was estradiol concentration on the skin. Skin estradiol levels were assessed at 10 min, 30 min, 60 min, and 120 min post-application.

**Results:**

The results indicated that the estradiol levels in the skin of the estradiol gel group were 205.29 ± 79.33, 193.64 ± 61.17, 99.15 ± 37.34, and 110.83 ± 69.81 at 10 min, 30 min, 60 min, and 120 min, respectively. In contrast, the estradiol content in the skin of the physical contact group was significantly lower, with levels of 65.87 ± 32.75, 59.06 ± 24.99, 7.95 ± 4.89, and 12.09 ± 3.71 at the same time points. Estradiol contamination was detected in all participants in the physical contact group; however, the levels were markedly lower than those in the estradiol gel group. In the estradiol gel group, estradiol levels remained stable within the first 30 min (p >0.05), rapidly decreased at 60 min (p <0.001) and remained stable from 60 min to 120 min (p >0.05). The trend in skin estrogen concentration over time in the physical contact group was consistent with that in the estradiol gel group.

**Conclusion:**

The study concludes that physical contact following application of estradiol gel can lead to skin contamination. Therefore, it is recommended that patients avoid skin exposure for at least 60 min after applying estradiol gel and refrain from physical contact with others, especially infants, children, individuals with breast cancer or other sex hormone-dependent tumors, and pets to minimize the risk of estradiol transfer.

## Introduction

Estrogen is widely used in clinical treatments such as menopause hormone therapy (MHT), assisted reproduction, and premature ovarian failure (1). Previously, oral estrogen was the first choice, but oral estrogen can increase the risk of thrombosis and gallstones due to its stimulation of the liver after intestinal absorption. Therefore, oral estrogen is not suitable for patients with high-risk factors for thrombosis and biliary stones (2). Estradiol gel is a new topical estrogen absorbed by the skin. It is colorless, and its main component is 17 β-Estradiol (3). It has similar efficacy as oral drugs in reducing menopausal symptoms (4, 5). Meanwhile, in the absence of intestinal absorption, there was no first-pass stimulation in the liver. Therefore, estradiol gel can effectively reduce the risk of venous thromboembolism (6), and is now widely used.

The instructions for estradiol gel recommend that the drug be applied to a large area of skin, such as arms, hips, waist, abdomen, and thighs to facilitate the absorption of estradiol. However, the relative bioavailability of the gel was only 61% compared to that of the tablets; thus, much of estradiol the remained on the skin (7). Physical contact with family members is inevitable in daily life. We believe that the estradiol residue on the patient’s skin poses a high risk of contamination in children, pets, men, and others through physical contact. It has been confirmed that exogenous estrogen can cause precocious puberty in girls and boys, which could cause serious damage to children’s health (8). Experiments have shown that estrogen can cause various male reproductive system development disorders, including gonadal dysplasia, testicular atrophy, decreased sperm quality, reduced sexual desire, and infertility (9–11). Therefore, the level of estradiol gel remaining after use is of great concern.

Once absorbed, residual estradiol on the skin diminishes, potentially decreasing the amount of estradiol transferred through physical contact. Therefore, we speculate that estradiol pollution may be time-dependent, and we hope to explore the degree of pollution at different medication times. In this study, we recruited 40 postmenopausal women who required hormone replacement therapy (HRT) with estradiol gel and 40 who did not use estrogen. After 10 min, 30 min, 60 min, and 120 min of estradiol gel application, a mimic physical contact was conducted, and we tested the skin estradiol level in the drug use and physical contact groups. By comparing the residual concentration of estradiol between the two groups at different times, we found an estradiol contamination curve, which provided an accurate data source for estradiol gel medication.

## Material and methods

In this study, we enrolled 40 menopausal women aged 40–60 years who needed MHT as the estradiol gel group and 40 women aged 20–45 years who did not use estradiol as the physical contact group at Changsha Maternal and Child Health Hospital Affiliated to Hunan Normal University between 2021 and 2022. Patients with sex-dependent tumors such as breast cancer, endometrial cancer, ovarian cancer, or uterine fibroids larger than 3 cm were excluded.

To ensure that the skin in the physical contact group was free of any residual estrogen, the forearms of the women in this group were fully cleaned by surgical washing before physical contact and dried. After washing, we used a cotton swab to sample the forearms and stored then in a dry specimen bottle as pre-contact data (n = 40) (**Supplementary Table 1**).

**Figure 1:**
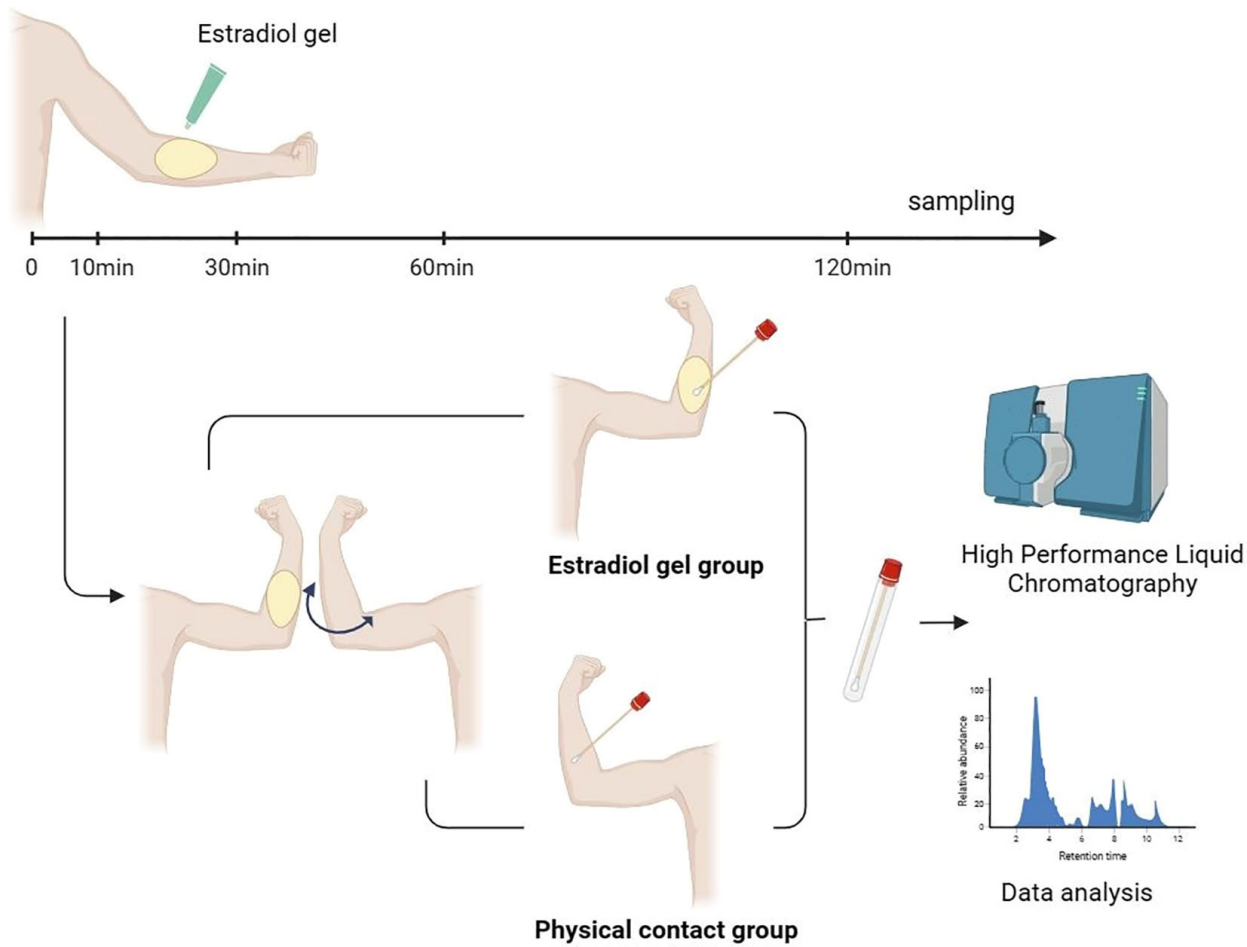
Flow chart investigating the contamination of the transdermal estrogen gel to the exposed person after physical contact. Estradiol gel was applied to forearm skin according to the manufacturer’s instructions. After skin dried, mimic physical contact was performed at 10 min, 30 min, 60 min, and 120 min, and the skin was sampled using a cotton swab. The sample was analyzed using liquid chromatography coupled with high-performance liquid chromatography (HPLC) and tandem mass spectrometry (MS/MS) to detect and quantify the presence of estradiol.

Patients in the estradiol gel group were evenly applied with a dosage of 2.5 g 17-β estradiol gel (contains 1.5 mg estradiol, produced by Wuhan Jianmin Group Suizhou Pharmaceutical Co., Ltd.) on the forearm skin. Estradiol gel was supplied with a standardized caliper designed to measure the precise amount of medication. The caliper employs varying lengths to control dosages, with standard measurements of either 2.5 g or 1.25 g. Initially, the gel was dispensed onto a caliper to achieve a length of 2.5 g, which contains 1.5 mg of estradiol. Subsequently, the gel was uniformly applied from the caliper to a 5 cm × 20 cm area on the forearm. Approximately 2 min later, the skin of these women was dry, and we divided them into four subgroups equally and mimicked physical contact at 10 min, 30 min, 60 min, and 120 min after the use of estradiol gel. In the physical contact group, the arm was rubbed horizontally and vertically at least 15 times with gel applied to the forearm of the estradiol gel group. Contact lasted for at least 30 s. Subsequently, a cotton swab was used to collect a sample from the drug application area, which was deposited in a dry specimen bottle. Estradiol levels in all 120 samples were quantified using high-performance liquid chromatography (HPLC) with a Shimadzu LC-20AD liquid chromatograph (**Figure 1**).

We employed SPSS (version 25.0; IBM) for statistical analysis and depicted the measured data as mean ± standard deviation (x ± s). For categorical variables, we used parametric independent-sample Student’s t-tests or non-parametric Mann–Whitney U tests and chi-squared tests when comparing rates between groups. When the data did not meet the requirement of the chi-squared test, we used Fisher’s exact test. Statistical significance was set at P <0.05.

## Results

Before conducting the formal study, we recruited 54 patients using estrogen gel and conducted a questionnaire survey. The results showed that 64.81% of participants were worried about the possibility of skin-residual drugs contaminating their relatives, and 40.74% stated that they had come into contact with their families after applying the drug. Of these relatives, 50% were children, 87.04% were husbands, and 37.04% were other family members.

**Figure 2:**
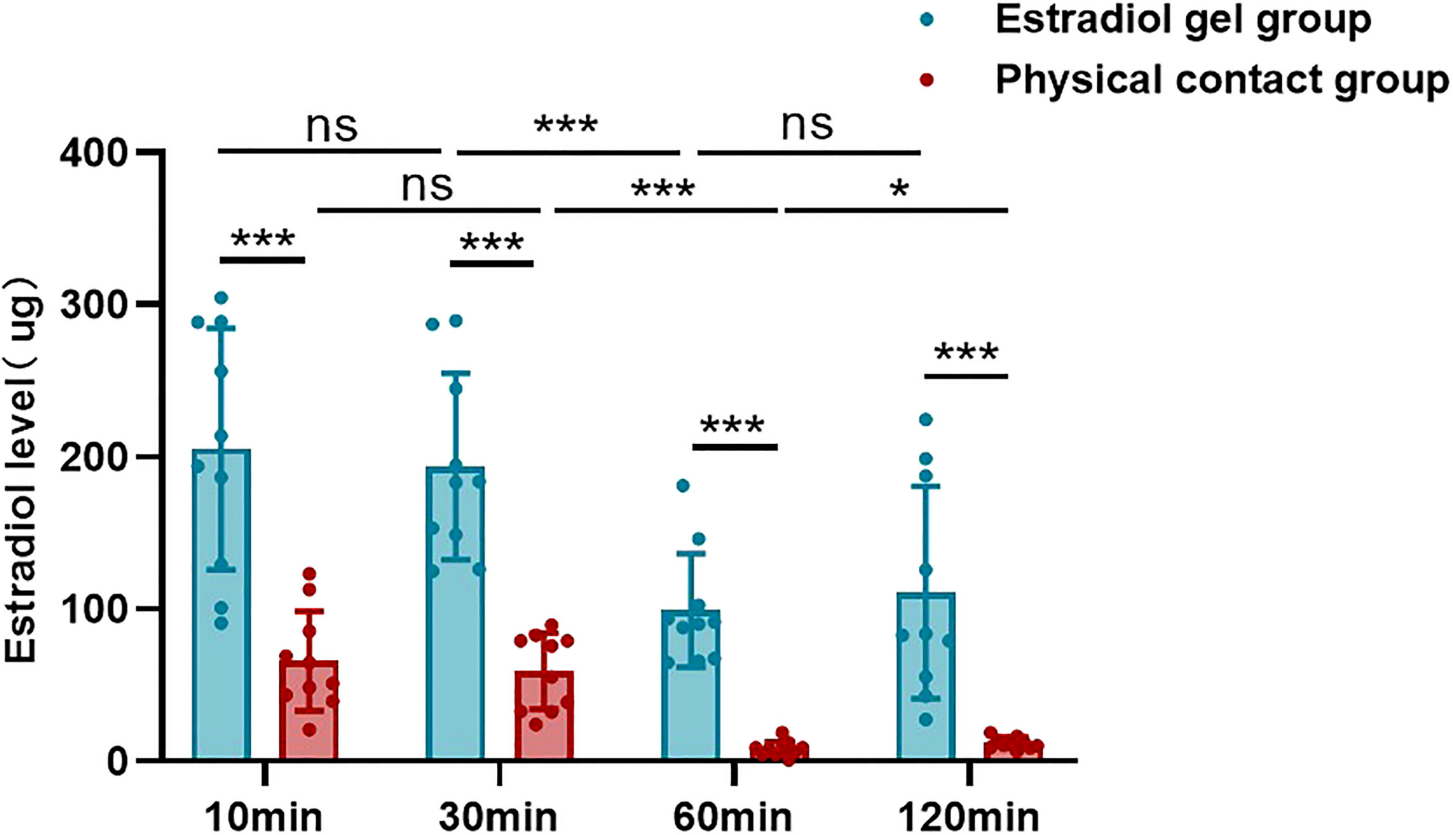
Estradiol levels in the skin of the estradiol gel and physical contact groups at different time points after gel application. “ns” represents p-value does not reach statistically different, “***” represents p <0.001, “*” represents p <0.05.

At 10 min, 30 min, 60 min, and 120 min after application, the skin estradiol concentration of the physical contact group was significantly lower than that of the estradiol gel group (205.29 ± 79.33 vs 65.87 ± 32.75, p <0.05; 193.64 ± 61.17 vs 59.06 ± 24.99, p <0.05; 99.15 ± 37.34 vs 7.95 ± 4.89, p <0.05; 110.83 ± 69.81 vs 12.09 ± 3.71, p <0.05), and the difference was statistically significant (**Table 1** and **Figure 2**).

It can be seen from the comparison of different treatment time that the skin estradiol concentration in the estradiol gel group remained stable within 10 min and 30 min (205.29 ± 79.33 vs 193.64 ± 61.17, p >0.05), decreased rapidly from 30 mi to 60 min (193.64 ± 61.17 vs 99.15 ± 37.34, p <0.001), and maintained a relatively stable level from 60 min to 120 min (99.15 ± 37.34 vs 110.83 ± 69.81, p >0.05) (**Table 1** and **Figure 2**). The physical contact group showed the same trend. There was no statistical difference in the skin estradiol content between 10 min and 30 min (65.87 ± 32.75 vs 59.06 ± 24.99, p >0.05), and the 60 min group showed a significant decrease compared with the 30 min (59.06 ± 24.99 vs 7.95 ± 4.89, p <0.05). The 120 min group basically maintained a lower level compared with the 60 min group (7.95 ± 4.89 vs 12.09 ± 3.71, p >0.05) (**Table 1** and **Figure 2**).

**Table 1:** Skin estradiol level in estradiol gel group and physical contact group at different time past gel application.

	10 min n = 10	30 min n = 10	60 min n = 10	120 min n = 10
Estradiol gel group n = 40	205.29 ± 79.33^a^	193.64 ± 61.17^be^	99.15 ± 37.34^ce^	110.83 ± 69.81^d^
Physical contact group n = 40	65.87 ± 32.75^a^	59.06 ± 24.99^bf^	7.95 ± 4.89^cfg^	12.09 ± 3.71^dg^

Data are presented as mean ± standard deviation, the unit of estradiol level is μg. The same letter indicates a statistical difference between the two groups.

**Figure 3:**
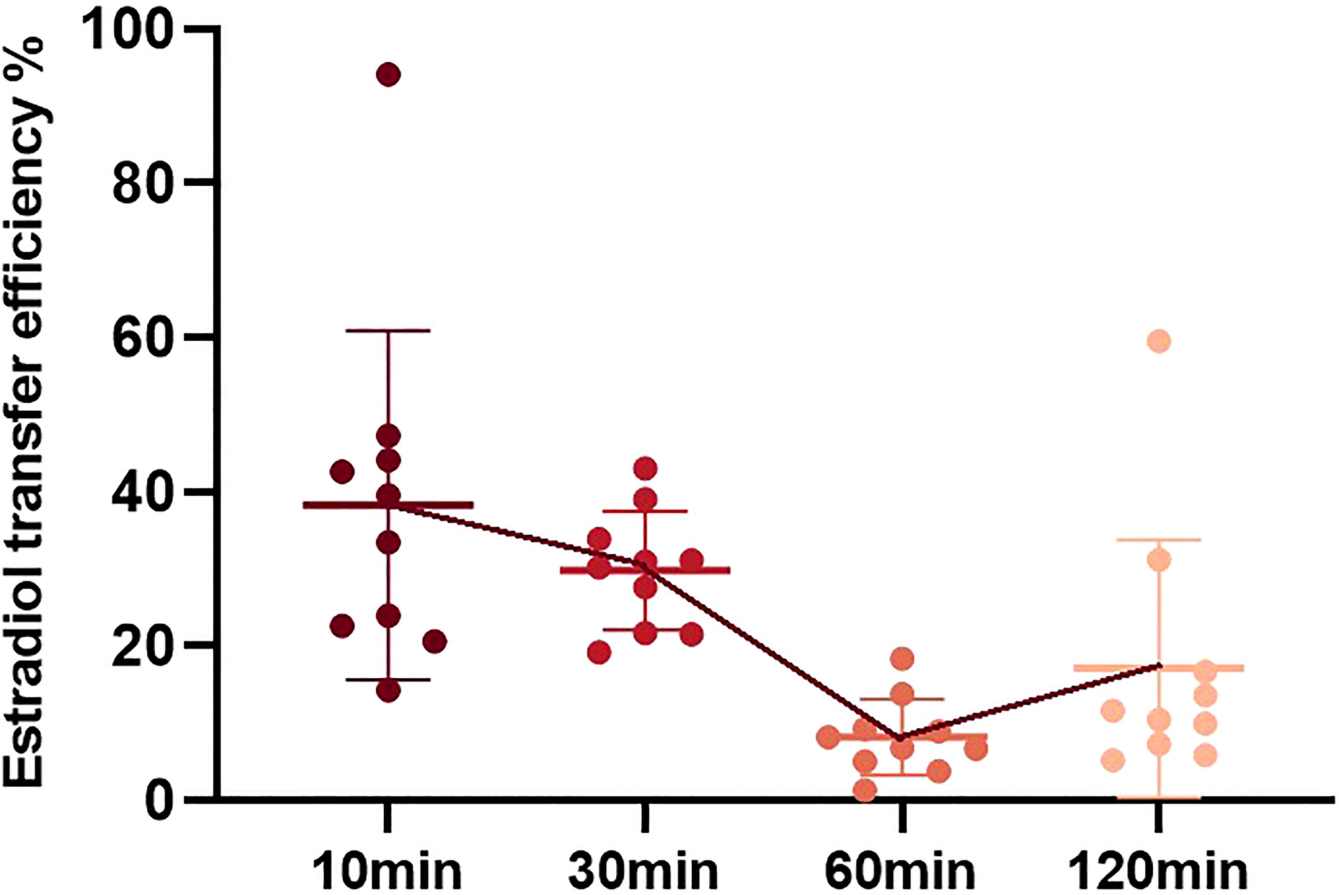
Estradiol transfer efficiency during physical contact at different time points after gel application. Estradiol transfer efficiency is defined as the estradiol amount in the simulated contact arm/estradiol amount in the medication female arm * 100%.

Taking each physical contact as an experiment, the transfer efficiency from the medicated woman to her contactor was different. Estradiol transfer efficiency was calculated as the percentage ratio of estradiol concentration in the simulated contact arm to that in the medicated female arm. Comparing the transfer efficiency of estradiol at different times, the average transfer efficiencies were 35.65% at 10 min, 29.82% at 30 min, 8.24% at 60 min, and 17.15% at 120 min (**Figure 3**). Comparing the data of the four groups, it can be concluded that the estradiol transfer efficiency decreased slightly at 10 min and 30 min, but there was no statistical difference. It decreased significantly after 60 min and increased slightly after 120 min.

## Discussion

Menopause is a natural physiological process that every woman must experience; however, the rapid reduction in hormone levels has brought a greater burden to women’s health. Menopausal hormone therapy (MHT) is a method that can relieve discomfort during the perimenopausal and postmenopausal period, and has attracted increasing attention from the public (12). MHT can improve menopause-associated symptoms, including vasomotor symptoms, genitourinary issues, sleep disturbances, and menopause-related anxiety, depressive symptoms, and arthralgia, it can also reduce menopause-related bone loss, lowers the risk of fragility fractures in older women, and reduces the incidence of self-reported diabetes (13). Experts have reached a consensus that MHT should begin at <60 years of age or <10 years of menopause (14). Therefore, the medication duration can be as short as 1–2 years and as long as 30–40 years for patients experiencing premature ovarian failure.

Although oral drugs are convenient for the long-term administration of estrogen, increasing risks of thrombosis and gallstones are inevitable (15). Without intestinal absorption, percutaneous estradiol has no first-pass liver stimulation effect, which greatly reduces the related risk. Guidelines recommend that percutaneous estradiol is more suitable for patients with VTE risk, hypertension, hypertriglyceridemia, obesity, diabetes, or a history of gallbladder disease (16–19). Owing to the lack of an inferior therapeutic effect to that of oral drugs, the wider applications of estradiol gel are being widely used in clinical treatments.

Gel preparation has several shortcomings. Daily application to the skin readily leads to secondary pollution in those in close physical contact, making it a concern in everyday life. Unlike the drugs used for short-term treatment, MHT requires years of administration, resulting in potential pollution that may persist for an extended period. Prolonged exposure to environmental estrogens may lead to irreversible health effects, including an increased risk of diseases such as breast cancer. Therefore, mitigating pollution is a crucial issue that merits further research.

What impelled us to undertake this study was the reports we received from two patients in our clinical practice. A 53-year-old menopausal woman discovered that her 8-month-old granddaughter developed breast development after approximately 1 year of applying estradiol gel. The menopausal woman was the actual caregiver of the infant. The second case involved a 36-year-old woman with premature ovarian failure who experienced breast development in her 3-year-old daughter after using estradiol gel. After the mother discontinued the drug, the breast development of the 3-year-old child returned to normal. We contend that these two instances of premature puberty in infants might be associated with the use of skin estrogen gel by caregivers, thereby leading us to complete this study.

Our study assumed that the degree of estradiol pollution through physical contact after different application times differed. Based on this, we explored the level of skin estrogen in contactors within 10 min–120 min after gel application and found that 60 min may be a key point. The application of estradiol gel to the skin causes estrogen contamination through physical contact. Contamination levels reached their peak 10 min after medication administration and subsequently began to decline; a significant decrease occurred at 60 min, and then remained stable until 120 min.

Wiener et al. reported that five pet dogs developed alopecia after close contact with their owners who had a history of estradiol gel use. When exposure was stopped, the hair of these dogs grew again. This suggests that estradiol gel pollution may cause canine alopecia (20). To the best of our knowledge, this is one of the few studies of estradiol gel contamination. However, the study did not explore whether the administration time was related to the degree of alopecia.

Our study indicated that estradiol pollution significantly decreased within 60 min of medication administration, potentially linked to the estradiol absorption rate. A study published in *Fertility and Sterility* administered estradiol gel to ovariectomized rhesus monkeys and found that the serum E2 level reached its peak 60 min after administration (21). This could explain our observation that a higher and faster estradiol absorption rate results in less estradiol remaining on the skin, consequently leading to reduced contamination.

Our study, which simulated real-world application of estradiol gel, identified a critical time point for the decline in estrogen contamination levels, emphasizing the importance of understanding the potential for contact contamination and its implications for patient safety. It has great clinical application value, with a rigorous design and reliable data analysis. However, this study also did not consider the potential impact of environmental temperature and humidity, which could confound the research findings by influencing estradiol absorption. However, the study was carried out in a room with central air conditioning, room temperature was approximately 25°C and the humidity was approximately 50%–60% and remained stable for a year.

Based on the results of this study, we recommend that patients using estradiol gel should be physically protection after application, not expose the skin, or have physical contact with others for at least 60 min to prevent estradiol contamination, especially in infants, children, pets, and patients with breast cancer and other sex hormone-dependent tumors. At the same time, the instructions for estradiol gel should warn about this potential pollution.

Through the study of the degree of estradiol contamination in physical contactors at various times after the application of estradiol gel, this project disclosed the dependence between the contamination and the time of application and provided a basis for the utilization of estradiol gel.

## Data Availability

The raw data supporting the conclusions of this article will be made available by the authors, without undue reservation.
