# Supplementary material for: Avoiding touching until 60 min—contamination of transdermal estradiol gel after physical contact
Source: Front Endocrinol (Lausanne). 2025 Jun 10;16:1524870. doi: 10.3389/fendo.2025.1524870 (PMC12185294; doi:10.3389/fendo.2025.1524870)
Supplement: Supplementary file 1 [file Table1.docx]

Supplement table 1 Basic skin estradiol level in physical contact group before contact.

| patient | 10min | 30min | 60min | 120min |
| --- | --- | --- | --- | --- |
| 1 | 0 | 3.78 | 0 | 0 |
| 2 | 0 | 0 | 0 | 1.65 |
| 3 | 0 | 10.12 | 0 | 2 |
| 4 | 0 | 0 | 3.06 | 0 |
| 5 | 0 | 0 | 0 | 0 |
| 6 | 0 | 0 | 0 | 1.3 |
| 7 | 0 | 0 | 0 | 0 |
| 8 | 4.9 | 0 | 0 | 0 |
| 9 | 0 | 0 | 0 | 0 |
| 10 | 0 | 0 | 0 | 0 |

Note:Estradiol level was displayed as μg.
